# Non-credible symptom report in the clinical evaluation of adult ADHD: development and initial validation of a new validity index embedded in the Conners’ adult ADHD rating scales

**DOI:** 10.1007/s00702-021-02318-y

**Published:** 2021-03-02

**Authors:** Miriam Becke, Lara Tucha, Matthias Weisbrod, Steffen Aschenbrenner, Oliver Tucha, Anselm B. M. Fuermaier

**Affiliations:** 1 Department of Clinical and Developmental Neuropsychology, Faculty of Behavioural and Social Sciences, University of Groningen, Grote Kruisstraat 2/1, 9712 TS Groningen, The Netherlands; 2 Department of Psychiatry and Psychotherapy, University Medical Center Rostock, Gehlsheimer Str. 20, 18147 Rostock, Germany; 3 Department of Psychiatry and Psychotherapy, SRH Clinic Karlsbad-Langensteinbach, 76307 Karlsbad, Germany; 4 Department of General Psychiatry, Center of Psychosocial Medicine, University of Heidelberg, 69115 Heidelberg, Germany; 5 Department of Clinical Psychology and Neuropsychology, SRH Clinic Karlsbad-Langensteinbach, 76307 Karlsbad, Germany

**Keywords:** Attention-deficit hyperactivity disorder, Conners’ adult ADHD rating scales, Feigning, Non-credible symptom report, Symptom validity

## Abstract

As attention-deficit/hyperactivity disorder (ADHD) is a feasible target for individuals aiming to procure stimulant medication or accommodations, there is a high clinical need for accurate assessment of adult ADHD. Proven falsifiability of commonly used diagnostic instruments is therefore of concern. The present study aimed to develop a new, ADHD-specific infrequency index to aid the detection of non-credible self-report. Disorder-specific adaptations of four detection strategies were embedded into the Conners’ Adult ADHD Rating Scales (CAARS) and tested for infrequency among credible neurotypical controls (*n* = 1001) and credible adults with ADHD (*n* = 100). The new index’ ability to detect instructed simulators (*n* = 242) and non-credible adults with ADHD (*n* = 22) was subsequently examined using ROC analyses. Applying a conservative cut-off score, the new index identified 30% of participants instructed to simulate ADHD while retaining a specificity of 98%. Items assessing supposed symptoms of ADHD proved most useful in distinguishing genuine patients with ADHD from simulators, whereas inquiries into unusual symptom combinations produced a small effect. The CAARS Infrequency Index (CII) outperformed the new infrequency index in terms of sensitivity (46%), but not overall classification accuracy as determined in ROC analyses. Neither the new infrequency index nor the CII detected non-credible adults diagnosed with ADHD with adequate accuracy. In contrast, both infrequency indices showed high classification accuracy when used to detect symptom over-report. Findings support the new indices’ utility as an adjunct measure in uncovering feigned ADHD, while underscoring the need to differentiate general over-reporting from specific forms of feigning.

**Supplementary Information:**

The online version contains supplementary material available at 10.1007/s00702-021-02318-y.

## Introduction

Growing recognition of adult manifestations of attention-deficit/hyperactivity disorder (ADHD) (Kessler et al. 2005a, b; Kessler et al. 2006; Simon et al. 2009; Wender et al. 2001) has drawn attention to diagnostic challenges inherent in the disorder’s clinical evaluation. As evidence of ADHD being an appealing and feasible target for exaggeration or feigning of symptom report has accumulated over the past years, the importance of identifying suspect effort within the diagnostic process has been underscored repeatedly (Fuermaier et al. 2016a, b; Fuermaier et al. 2017a, b; Harrison and Armstrong 2016; Harrison et al. 2007; Jachimowicz and Geiselman 2004; Lee Booksh et al. 2010; Marshall et al. 2016; Quinn 2003; Smith et al. 2017; Walls et al. 2017). However, this growing base of empirical evidence supporting the use of validity tests in the diagnostic work-up of ADHD does not appear to have found widespread application in clinical settings yet. Harrison et al. (2013) found less than a third of surveyed professionals to be knowledgeable about the empirical evidence supporting the use of symptom validity tests (SVTs) in the clinical assessment of ADHD. Shortly after, Nelson et al. (2014) published their review of psychological reports documenting ADHD evaluations and found that the use of SVTs was mentioned in 3% of examined reports. This mismatch between science and clinical practice may in part be due to professional guidelines having lacked information on the role of validity testing in the diagnostic process of ADHD until a short time ago (see for example Gallagher and Blader 2001, who mention incentives to feign ADHD as well as the possibility of bias in the evaluation process but not the use of specialized validity tests; Gibbins and Weiss 2007). The European Consensus Guidelines on the diagnosis and treatment of adult ADHD (Kooij et al. 2019), on the other hand, recently and explicitly endorsed the use of validity testing. Until such guidelines routinely inform clinical practice, concerns about the consequences of the failure to identify suspect symptom report remain. Repercussions of unjustified ADHD diagnoses concern both the individual under examination as well as society at large.

Courrégé et al. (2019) called attention to an unwarranted risk of medication side effects among those who have been wrongfully diagnosed with ADHD. Even though stimulant medication prescribed to alleviate symptoms of the disorder has failed to show consistent positive effects on cognitive functioning in neurotypical adults (Advokat 2010; Hall and Lucke 2010; however, see also Marraccini 2016), there is widespread belief in its ‘neuroenhancing’ effects (Bossaer et al. 2013; London-Nadeau et al. 2019; Rabiner 2013; Rabiner et al. 2009). Rising numbers of self-referrals (Hagar and Goldstein 2001; Harrison et al. 2008) and significant rates of illicit use or distribution of stimulant medication (Advokat et al. 2008; Bossaer et al. 2013; Low and Gendaszek 2002; Wilens et al. 2008) indicate how obtaining a prescription of such medication may act as a potent incentive motivating individuals to seek assessment and a diagnosis of ADHD.

Whereas those with a false positive diagnosis are potentially at an increased risk of adverse health effects due to superfluous treatment, much needed resources and accommodations may not be available to genuine patients with ADHD as a consequence of unwarranted diagnoses. Accommodations at school or work, including quiet work spaces and assisting technology, such as noise-cancelling headphones, may be scarce resources if utilized by individuals who have been wrongfully diagnosed with ADHD.

On a societal level, illegitimate diagnoses of ADHD may fuel public debates on whether the disorder is real. In a recent survey, Speerforck et al. (2019) found one-fifth of respondents to voice the belief ADHD was not a real disease. Amidst popular press releases speaking of an ‘ADHD epidemic’, rising numbers of newly diagnosed cases (Davidovitch et al. 2017) are often mentioned in the same breath as doubts about ADHD being a real disorder. Base rates of self-reported symptoms of ADHD are high indeed (DuPaul et al. 2001; Faraone and Biederman 2005; Harrison 2004; Heiligenstein et al. 1998; Lewandowski et al. 2008; McCann and Roy-Byrne 2004; Murphy and Barkley 1996; Weyandt et al. 1995), and Suhr et al. (2011) suggest that symptom exaggeration or feigning may partly account for the frequent occurrence of ADHD-like symptoms in the general population. Inclusion of such false positive cases of ADHD in treatment trials may further undermine public confidence in effective treatment options.

In light of a high clinical need for accurate assessments of ADHD, falsifiability of instruments commonly used in the diagnostic process is of concern. Since symptoms of ADHD are largely subjective, self-report questionnaires and structured interviews are essential tools in securing a diagnosis of adult ADHD. Yet studies conducted over the past years have repeatedly shown that these very instruments are inaccurate in the detection of non-credible symptom report (Harrison et al. 2007; Jachimowicz and Geiselman 2004; Booksh et al. 2010; Quinn 2003). Individuals who simulate or exaggerate their complaints frequently score in the plausible, clinical range on these instruments, and few scales or interviews include validity indicators. The few existing embedded validity indicators are oftentimes based on inconsistency rather than exaggeration of symptom report (e.g., Inconsistency Index embedded in the Conners’ Adult ADHD Rating Scales). As simulating individuals have been shown to obtain exaggerated high scores when compared to genuine cases of adult ADHD (Harrison et al. 2007), however, the latter strategy may be more suitable to detect non-credible symptom report in the assessment of ADHD.

By introducing an infrequency index to the Conners’ Adult ADHD Rating Scales (CAARS) (Conners et al. 1999), Suhr et al. (2011) were first to offer a possible solution to the unmet diagnostic need of assessing symptom over-report in ADHD. The CAARS Infrequency Index (CII) was developed by selecting only those original CAARS items, which were infrequently endorsed by healthy members of the general public and genuine patients with a secured diagnosis of ADHD. The resulting sum score showed utility in discerning genuine cases of ADHD from individuals who had failed an independent performance validity test (PVT; see “Methods” section for details). Subsequent cross-validations of this index have revealed variable, yet promising classification accuracy (Cook et al. 2016, 2017; Edmundson et al. 2017; Fuermaier et al. 2016a, b; Harrison and Armstrong 2016; Walls et al. 2017).

Harrison and Armstrong (2016) provided researchers and clinicians with an additional validity indicator embedded in the CAARS. Their Exaggeration Index (EI) combines items adapted from the Dissociative Experiences Scale (DES) (Bernstein and Putnam 1986), which are very rarely endorsed by non-clinical populations, with high scores on two CAARS DSM scales. The EI’s sensitivity to feigned ADHD spun from 24 to 69% and specificity ranged from 74 to 97%, depending on which cut score was applied. EI-items were not added to the CAARS version under examination here (see next section).

More recently, Courrégé et al. (2019) developed the ADHD Symptom Infrequency Scale (ASIS). Its Infrequency Scale (INF) includes items which were written for the explicit purpose of detecting symptoms rarely reported by genuine patients with ADHD. As such, the ASIS is the first instrument to include disorder-specific items developed for the detection of non-credible symptom report. The authors disclose that these new items were based on stereotypes of ADHD, but provide few details on the theoretical underpinnings which informed the development of these new items. Courrégé et al. (2019) present highly promising results with regard to the INF’s psychometric properties, and its classification accuracy in particular. The scale’s sensitivity in distinguishing genuine from simulated ADHD ranged from 79 to 86%. Specificity lay at 89%.

Similar to the ASIS’ infrequency scale, Robinson and Rogers (2018) based the development of their Dissimulation ADHD Scale (Ds-ADHD) on misconceptions about or erroneous stereotypes of ADHD. In contrast to the ASIS, the Ds-ADHD does not contain any newly written items. Instead, the authors asked participants without secured diagnoses of ADHD to indicate which items of the MMPI-2-RF (Ben-Porath and Tellegen 2008) they considered most relevant to identifying the disorder. Items were selected for the final scale if more than 50% of participants without ADHD deemed them characteristic of the disorder and more than 50% of patients with ADHD marked them as not applicable (i.e., ‘false’). Ten MMPI-items remained. Responses to these items were recoded and summed up to form a 10- to 20-point scale. The authors reported very large effect sizes for the Ds-ADHD when distinguishing simulating participants from adults with a secured diagnosis of ADHD (*d* = 1.84) and examinees feigning general psychological disorders (*d* = 2.65). Sensitivity of the Ds-ADHD in detecting feigned ADHD was 75%, specificity was 97%.

Robinson and Rogers (2018) conclude their study by recommending the use of erroneous stereotypes in the development of disorder-specific validity tests. Adaptation of detection strategies previously described by Rogers (2018) may be useful additions or alternatives to the reliance on erroneous stereotypes, according to the authors. They suggest that inquiries into symptom combinations, which are rarely reported jointly by genuine patients, may present another basis on which to formulate ADHD-specific items for validity tests; a recommendation supported by findings published recently (Becke et al. 2019).

The present study aimed to develop new ADHD-specific infrequency items by adapting detection strategies formulated by Rogers (2018), including symptom combinations and supposed symptoms (see “Methods”). Initial data on the utility of the new index in distinguishing genuine adult ADHD from non-credible presentations were collected using a simulation design. In addition to instructed simulators’ responses, we examined the new index’ ability to identify patients with secured diagnoses of ADHD who had failed an independent performance validity test.

## Methods

### Participants

*Neurotypical Control Group* The *Neurotypical Control Group* was recruited from a pool of panel members registered with a Dutch online platform. This website invites interested members of the public to take part in online studies in exchange for financial reward. Invitations to partake in the present study were accepted by 1577 adults from the Netherlands. Reconcilable with an average drop-out rate of 30% reported for online studies (Galešić 2006), 460 adults (29.17%) withdrew from participation before they had completed all instruments examined as part of the present study. They were consequently excluded from further analyses due to missing data. Similarly, 35 volunteers in this group (2.22%) left five or more CAARS items unanswered. In accordance with the scales’ manual (Conners et al. 1999), these protocols were dismissed as invalid. Eighteen additional participants (1.14%) were excluded due to neurological or psychiatric comorbidities, or recent intake of medications known to affect the central nervous system (*n* = 45, 2.85%).

Eighteen participants in the control group (1.14%) presented with significantly elevated *T*-Scores on at least one DSM Scale of the CAARS. *T*-Scores equal to or above 80 are expected to occur very infrequently among honest-responding, healthy adults who exert adequate effort during testing and are thus considered indicative of non-credible responding by the instruments’ authors (Conners et al. 1999). We therefore removed these 18 controls from the pool of credible control participants and summarized them in an *Overreporting Control Group* instead. Their data were not considered in the development of the new infrequency index, but served in its initial validation.

The median age of the 1001 remaining credible controls equaled 49 years, with a *range* of 40 years and a median absolute deviation (MAD) of 10 years (minimum = 25, maximum = 65). The number of male (*n* = 494, 49.40%) and female (*n* = 504, 50.30%) participants was balanced. Three volunteers (0.30%) did not disclose their gender. Participants in this group reported an average of 13 years spent in education (MAD = 3). Table 1 provides a summary of all descriptive data.

**Table 1:** Descriptive data by group

	Neurotypical Control Group		ADHD Group		Simulation Group
	Credible	Overreporting	Credible	Non-Credible	
*n*	1001	18	100	22	242
Total	1019		122		
Age (years)					
Median (MAD)	49 (11)	32 (4)	34 (9)	31.50 (10.5)	20 (1)
Range	40	33	62	42	42
Sex (m/f)	494/504	13/5	46/54	13/9	64/178
%	49.4/50.3*	72.2/27.8	46.0/54.0	59.1/40.9	26.4/73.6
Education					
Years					
Median (MAD)	13 (3)	13 (3)	13 (3)	14 (2)	13 (1)
Range	10	10	16	15	15
ADHD symptomatology					
Past^a^					
Median (MAD)			40.0 (10)	40.5 (14.5)	14.0 (7)
Range			70	53.5	56
Present^b^					
Median (MAD)			31.0 (6)	28.5 (5.5)	11.0 (5)
Range			53	40	45

*MAD* median absolute deviation

^a^Wender Utah Rating Scale

^b^ADHD Self-Report Scale

^*^Three participants did not disclose their gender

*Overreporting Controls* were significantly younger (Md = 32, MAD = 4) than *Credible Controls* (*z* = 3.341, adjusted *p* = 0.008) and more commonly male (72.20%) than female (27.80%). Gender distribution therefore differed between credible and over-reporting controls, though the difference did not reach statistical significance [*χ*^*2*^ (2) = 3.717, *p* = 0.156]. The groups were comparable with regard to their years spent in formal education (*z* = − 0.733, adjusted *p* = 1.00; see also Table 1).

*ADHD Groups* One-hundred-and-thirty-three adults with ADHD, who had been referred to the Department of Psychiatry and Psychotherapy at the SHR Clinic in Karlsbad-Langensteinbach, Germany, by local psychiatrists or neurologists, took part in the study. Diagnoses of ADHD were secured through a comprehensive clinical work-up and confirmed by at least two experienced clinicians. The diagnostic process included a psychiatric interview, which enquired both past and present symptoms of ADHD in accordance with the DSM criteria (American Psychiatric Association 2013; Barkley and Murphy 1998). Additionally, participants scored above the recommended cut-offs on two standardized self-report rating scales, which tapped symptoms of ADHD across the same time span (WURS-K and ASR) (Adler et al. 2006; Kessler et al. 2005a, b; Ward et al. 1993). Their reports were further corroborated through external records identifying objective impairments in line with the diagnosis of ADHD, such as struggle in school or employment. Wherever possible, inquiries were posed to multiple informants (e.g., evaluations made by employer alongside reports of parents or partners). Lastly, validity of participants’ test performance was examined by means of the Test of Memory Malingering (TOMM) (Tombaugh 1996) or the Groningen Effort Test (GET) (Fuermaier et al. 2016a, b, 2017a) in cases for whom the TOMM was not available. Twenty-two participants scored above the recommended cut-off scores on the TOMM (*n* = 5, 3.76%) or the GET (*n* = 17, 12.78%) and were thus removed from the pool of credible patients. Like *Overreporting Controls,* these *Non-Credible Patients* were excluded from the development of the new index. Four adults with ADHD (3.01%) were excluded as they completed neither the TOMM nor the GET. Seven additional patients with ADHD were excluded due to missing (*n* = 6, 4.51%) or incomplete (*n* = 1, 0.75%) data on the CAARS. In total, 100 participants remained in the *Credible ADHD Group* and 22 individuals formed the *Non-Credible ADHD Group*.

The *Credible ADHD Group* differed significantly from *Credible Controls* in age (MD = 34, MAD = 9, Range = 62; *z* = *− *7.255, adjusted *p* < 0.01), but not gender distribution (*χ*^*2*^ (2) = 0.747, *p* = 0.688) or education (*z* = 1.572, adjusted *p* = 1.00). Compared to *Overreporting Controls*, the *Credible ADHD* included a greater percentage of female participants [*χ*^*2*^ (2) = 4.196, *p* = 0.041]. Age (*z* = 0.132, adjusted *p* = 1.00) and education (*z* = 0.958, adjusted *p* = 1.00) did not differ between the groups.

Descriptive data of both credible and non-credible adults with ADHD are presented in Table 1. The credible and non-credible patient groups were comparable with regard to age (*z* = 0.294*,* adjusted *p* = 1.00), gender distribution [*χ*^*2*^ (1) = 1.237, *p* = 0.266], and education (*z* = − 1.893, adjusted *p* = 0.584). Both credible and non-credible patients with ADHD most commonly met diagnostic criteria for the combined subtype (*Credible ADHD Group*: 48%; *Non-Credible ADHD Group*: 68%), with the inattentive subtype being less common (*Credible ADHD Group:* 42%; *Non-Credible ADHD Group*: 27%). Two credible patients (2%) had been given a diagnosis of the hyperactive subtype, whereas no subtype was specified for nine cases (9%). Sixty-two percent of participants in the *Credible ADHD Group* reported psychiatric or neurological comorbidities, most commonly mood (*n* = 46) or anxiety (*n* = 16) disorders (see Appendix 1 in ESM for an overview of all diagnoses). Occurrence of more than one comorbid disorder was common, with 21 participants having received two additional diagnoses alongside ADHD and five adults having been diagnosed with three or four comorbidities. A similar picture emerged among non-credible adults with ADHD, where 68% of participants reported at least one relevant comorbidity. Given the high prevalence of comorbidities among adults with ADHD (Biederman et al. 1993), participants in the *ADHD Groups* were *not* excluded from the current study if they reported such additional disorders.

*Simulation Group* A group of 260 adults was recruited through public announcements, researchers’ contacts, as well as word-of-mouth, and asked to feign ADHD throughout relevant portions of the study protocol (see “Procedure” for details). Three participants were excluded from further analyses due to missing (*n* = 2, 0.70%) or incomplete (*n* = 1, 0.35%) data on the CAARS. Fifteen simulators (5.28%) were excluded as they reported psychiatric disorders other than ADHD in the course of testing. Reported symptoms of ADHD (i.e. clinical elevations on WURS-K and ADHD-SB), on the other hand, were *not* considered a criterion justifying exclusion from the study.

As shown in Table 1, median age of the 242 remaining simulators was 20 years (MAD = 1, range = 42). They were thus younger than participants in all other groups (*p* < 0.01 in all cases). As the majority of simulating participants was female (*n* = 178, 73.60%), the gender distribution in this group also differed from the *Credible* (*χ*^*2*^ (1) = 42.625, *p* < 0.01) and *Overreporting Control Groups* (*χ*^*2*^ (1) = 16.842, *p* < 0.01) as well as the *ADHD Groups* (*χ*^*2*^ (1) = 12.40, *p* < 0.01 for the comparison with credible patients; *χ*^*2*^ (1) = 10.402, *p* = 0.01 when comparing simulators to non-credible patients). In terms of education, simulators differed from credible participants in the control group (*z* = − 8.446, adjusted *p* < 0.001) and the patient group (*z* = − 3.583, adjusted *p* = 0.003), but not from over-reporting controls (z = 0.078, adjusted *p* = 778) or non-credible patients with ADHD (*z* = 0.896, adjusted *p* = 1.00).

### Materials

*ADHD Symptom Severity* Severity of both past and present ADHD symptomatology was assessed by means of self-report. Childhood symptoms of ADHD were measured using the Wender Utah Rating Scale (WURS-K) (Ward et al. 1993). Its short from taps ADHD symptomatology experienced between the ages of eight and ten years on 25 items, which are rated on a five-point scale. Response options range from 0 (‘Dos not apply’) to 4 (‘Strong manifestation’). A total score is obtained by summing up all items except numbers 4, 12, 14, and 25. If the resulting sum score exceeds the recommended cut-off value of 30, symptoms are presumed to have been clinically significant.

Current ADHD symptomatology was assessed by means of the ADHD self-report scale (ASR) (Adler et al. 2006; Kessler et al. 2005a, b). Its 18 items enquire symptoms of ADHD as described in the DSM-IV (American Psychiatric Association 2000). Participants indicate their answer on a four-point scale ranging from 0 (‘Does not apply’) to 3 (‘Strong manifestation’). The sum of all item scores represents the total score, which is assumed to be indicative of clinically relevant symptoms if it surpasses the cut-off score of 18.

*Conners’ Adult ADHD Rating Scale (CAARS)* In their long form, the Conners’ Adult ADHD Rating Scales (CAARS) (Conners et al. 1999) are a 66-item self-report measure intended to quantify presence and severity of ADHD symptomatology. Participants are presented with statements pertaining to everyday activities and tendencies in behavior, and asked to indicate the extent to which they are applicable. All items are rated on a four-point scale, ranging from 0 (‘not at all/never’) to 3 (‘very much/very frequently’). Sum scores are calculated for nine subscales, with higher scores indicating increasing symptom levels. Subscales include factor-derived scales assessing inattention and memory problems, hyperactivity and restlessness, impulsivity and emotional lability, as well as participants’ self-concept. Three scales measure ADHD symptoms as listed in the DSM-IV (American Psychiatric Association 2000), and an additional score summarizes these scales in a DSM Total.

Twelve CAARS items, which best distinguish adults with ADHD from their non-clinical counterparts, form the ADHD Index. No specific cut-off score is recommended for this index, but individuals with *T*-values above 70–75 likely meet the diagnostic criteria of ADHD. *T*-scores above 80 should be considered indicators of severe symptomatology or possible non-credible responding, according to the authors (Conners et al. 1999). They report a sensitivity of 87% and a specificity of 85% for the ADHD Index. The CAARS further includes an Inconsistency Index intended to uncover careless or random responding. Participants’ responses are considered suspect if their scores on this index exceed eight.

As touched upon previous sections, additional indices were later embedded in the CAARS to aid the detection of non-credible self-report. Suhr et al. (2011) introduced the CAARS Infrequency Index (CII) while Harrison and Armstrong (2016) developed the Exaggeration Index (EI). The CII’s development was based on a rare symptoms approach using the CAARS’ original items. The authors selected those items for the CII, which were endorsed by no more than 10% of healthy controls and adults with ADHD. Twelve items met these criteria and responses to these items were summed up to form the CII score. Herein, the instruments’ original four-point scale was retained. High endorsement of multiple rare symptoms included in the CII was assumed to be indicative of exaggerated responding, and sum scores exceeding 20 were considered suspect. Using this cut score, the CII’s initial validation supported its use in the detection of non-credible responding in the diagnostic process of ADHD. The authors report a sensitivity of 24% and a specificity of 95% when using the CII as a criterion in distinguishing genuine cases of ADHD from individuals who failed an independent performance validity test. Subsequent cross-validations revealed varying classification accuracy of the CII, with sensitivity estimates ranging from 17% or 18% (Cook et al. 2017,2016), 34% (Walls et al. 2017) to approximately 50% (Fuermaier et al. 2016a, b; Robinson and Rogers 2018). Specificity of the CII has been found to be high, ranging from 86% (Robinson and Rogers 2018) to 95% (Walls et al. 2017; see Fuermaier et al. 2016a, b for an exception).

*Experimental Version of the CAARS (CAARS-ACI)* As part of this study, 15 new items were introduced to the long form of the CAARS. We denote this expanded version CAARS-ACI to allude to the new infrequency index: the ADHD Credibility Index (ACI).

The newly written items represented disorder-specific adaptions of detection strategies used in existing tests of malingering and deception (see Rogers 2008). Five items in the CAARS-ACI aim to identify non-credible responding by presenting examinees with highly selective symptom reports. Symptoms described in these items are unrealistically precise, for instance, enquiring about inattention manifesting only during specific times of the week. Four items examine supposed symptoms, which may reflect lay persons’ impressions of or public misconceptions about ADHD (e.g., reports of having received too little parental attention in childhood). Exaggerated symptoms and implausible symptom combinations are tapped by three items each. While examples of the former enquire, for example, about reports of extremely disorganized home environments, the latter ask examinees about fidgety behavior shown to attract others’ attention. The new items were distributed randomly among the original CAARS items and rated on the same four-point scale. They are not presented as part of this paper to ensure test security, yet they are available from the authors upon reasonable request.

*Tests of Performance Validity in ADHD Groups* Credibility of patients’ performance during testing was examined using the Test of Memory Malingering (TOMM) (Tombaugh, 1996) or the Groningen Effort Test (GET) (Fuermaier et al. 2017a, b, 2016a, b) to ensure that the *ADHD Group* would include genuine cases only.

First introduced in 1996, the TOMM is a visual memory recognition test which utilizes a forced-choice format and floor effects to detect non-credible symptom reports. If participants identified fewer than 45 of 50 items correctly on Trials 1 or 2, their performance was considered suspect and they were excluded from the *credible ADHD Group*. Given this cut-off value, the TOMM’s sensitivity amounts to 56% and its specificity to 93% (Greve et al. 2006).

The GET is a computerized test developed to uncover non-credible performance during the diagnostic process of ADHD. It confronts participants with a visual discrimination task designed to appear cognitively taxing, with high demands on attention and concentration. Unbeknownst to examinees, however, most individuals—including those with ADHD—complete the task with ease. A cut-off score allows for the discrimination of credible and non-credible performance with a high degree of accuracy: the GET’s sensitivity and specificity have been reported at 89% (Fuermaier et al. 2017a, b).

### Procedure

*Neurotypical Control Group* The assessment procedure for healthy participants was approved by the Ethical Committee Psychology (ECP) at the University of Groningen. All participants in the *Control Group* gave written informed consent and were subsequently asked for anamnestic information including age, sex, and educational attainment. Additionally, participants were asked about any history of psychiatric or neurological disease, as well as pharmacological treatments affecting the central nervous system. They were then instructed to complete all self-report measures (i.e., WURS-K, ASR, CAARS-ACI) honestly and to the best of their ability.

*ADHD Groups* Having given informed consent, adults with ADHD were tested individually in a quiet room on clinic premises. They were assured that all data collected as part of the study would be analyzed anonymously and that the results would not affect their clinical assessment or treatment. No reward was offered for participation in the research project. Patients underwent a comprehensive clinical assessment, which encompassed self-report questionnaires, standardized measures of cognition, as well as the previously described validity tests. Testing took approximately 2 h, divided into two parts to avoid potential effects of fatigue (Lezak et al. 2004). The study complied with the ethical standards of the Helsinki Declaration and was approved by the local institutional ethical committee (Medical Faculty at the University of Heidelberg, Germany).

*Simulation Group* Like honest-responding controls, participants allocated to the *Simulation Group* gave written informed consent, provided anamnestic information, and completed a validity test. In contrast to the *Control Group*; however, they were asked to answer the CAARS-ACI as though they had ADHD. Examiners were aware of the instructions the simulating participants received.

To help them adopt the role of an adult with ADHD, participants in this group were provided with a vignette describing multiple possible incentives for someone to simulate the disorder (e.g., financial, educational or vocational accommodations, or the prescription of stimulant medication). Volunteers were explicitly asked to feign ADHD in a realistic manner by providing believable answers (i.e., avoiding pronounced exaggeration of symptoms). This was further incentivized by introducing the chance of winning a tablet PC if they were the one participant who feigned the condition most convincingly. In actuality, the PC was awarded to a randomly chosen participant; that is, irrespective of test performance. Following the assessment, which took approximately 70 min, participants were debriefed and instructed to stop feigning the disorder. Additionally, they were asked whether they had followed the given instructions. All participants answered in the affirmative.

### Statistical analyses

*Item Selection, Calculation of ADHD Credibility Index (ACI) Scores, and Determination of Cut-Off Score* In line with the approach first described by Suhr et al. (2011) in the development of the CAARS Infrequency Index (CII), items were selected for the new infrequency index—henceforth termed ADHD Credibility Index (ACI)—if they were endorsed by no more than 10% of the sample combining credible adults with ADHD and credible neurotypical controls. This approach was chosen to minimize the occurrence of false positive classifications (Suhr et al. 2011).

To allow for a dichotomous distinction between endorsed and non-endorsed items, responses given on the CAARS’ four-point scale were rescored, such that items endorsed with “0” or “1” were coded 0 (i.e. not endorsed). Responses of “2” or “3” were recoded as 1 (i.e., endorsed). Each participant’s score on the ADHD Credibility Index was calculated by summing up the scores on the new items which had been endorsed infrequently by patients with ADHD and control participants alike. Again, in accordance with the approach taken by Suhr et al. (2011), the CAARS’ initial four-point scale was used in this step. To find a cut-off score that maximized specificity, the distribution of scores was examined and a score determined below which at least 90% of participants of both non-simulating groups (i.e., *Credible Controls* and *Credible ADHD Group*) fell. Herein, we considered effects of both age and sex by conducting non-parametric significance tests of group differences and providing separate summary statistics on ACI scores.

*Association with Symptoms of ADHD* To examine whether symptoms of ADHD were associated with elevated scores on the ADHD Credibility Index, we considered *T*-Scores above 65 on the DSM Scales indicative of clinically relevant ADHD symptomatology. This diverges from the CAARS manual, which suggests *T*-Scores above 70 or 75 to signal relevant symptomatology, but allows for comparability with the CII (Suhr et al. 2011). We noted the percentage of individuals with such clinically elevated scores, who also showed suspect scores on the ADHD Credibility Index (ACI), as well as possible differences to those without elevations on the DSM Scales (i.e., *T*-Scores < 65).

*Utility of the ADHD Credibility Index in the detection of non-credible symptom report* The ADHD Credibility Index’ utility in discriminating genuine cases of adult ADHD from non-credible responding was examined in a series of ROC analyses.

*Simulation design and non-credible patient data* In a first step, we determined the ADHD Credibility Index’ ability to discern the *ADHD Group* from the *Simulation Group*. Second, the ACI was used as a criterion distinguishing *credible* from *non-credible* adults with ADHD (i.e. those who had failed either the TOMM or GET as independent validity measures). The same analyses were run using the CAARS’ DSM Scales and the CII as criteria, such that the ACI’s performance could be compared to that of suspect *T*-Score elevations and the CII.

*Concordance with existing validity indicators* We conducted a ROC analysis to investigate whether the ADHD Credibility Index was useful in detecting over-report on the DSM Scales (i.e., *T*-scores above 80) in the complete sample collapsed across groups. Agreement between the ACI and existing CAARS validity indicators was also determined. Specifically, the ADHD Credibility Index was compared to *T-*score elevations equal to or above 80 on the DSM Scales, to scores equal to or above 8 on the Inconsistency Index, and to suspect results (i.e., scores ≥ 21) on Suhr’s Infrequency Index (CII).

## Results

### Item selection, calculation of ADHD credibility index scores, and determination of a cut-off score

**Fig. 1:**
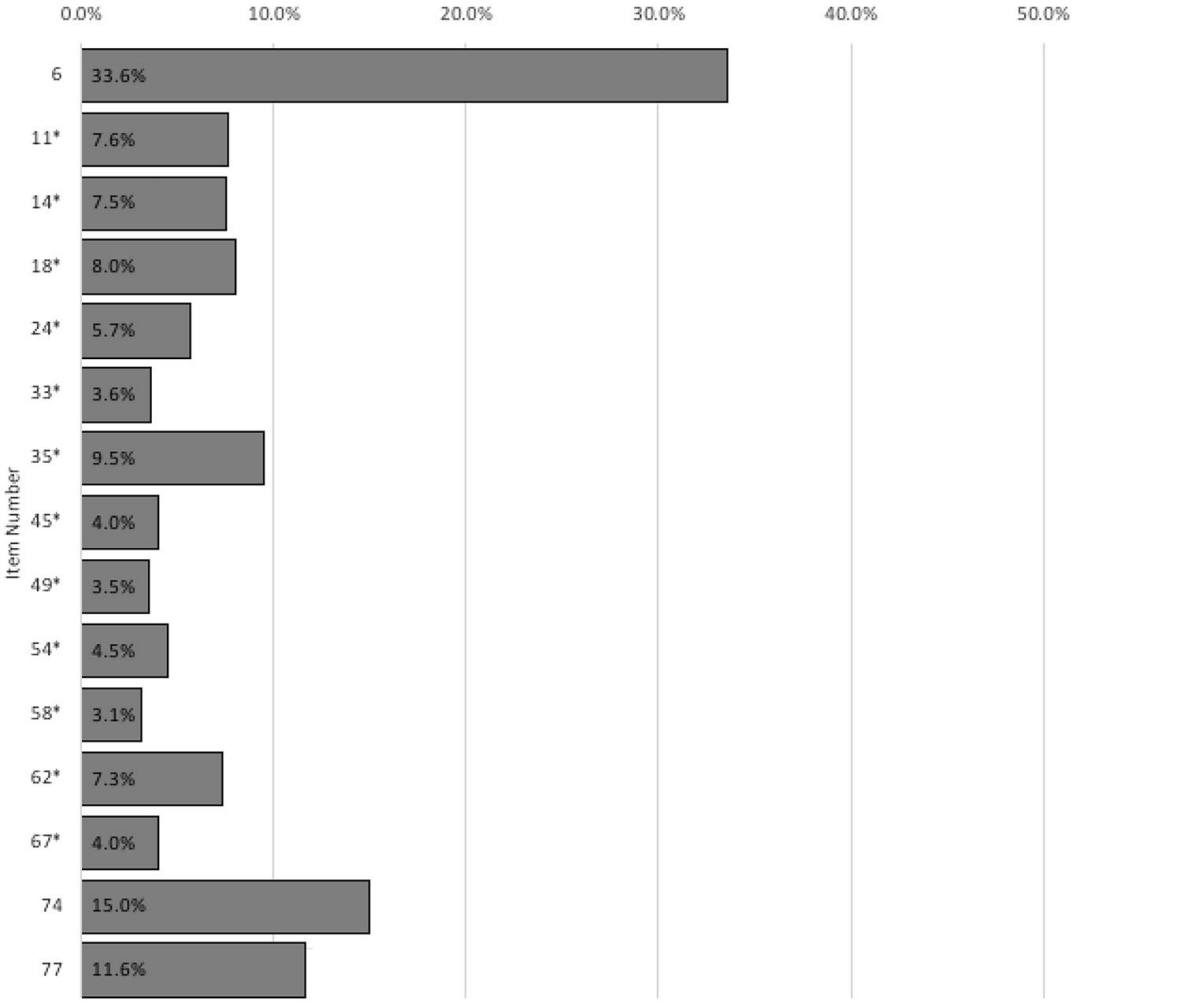
Endorsement of New Items by Participants in the *Neurotypical Control Group* and *ADHD Group*. *Note*. Bars illustrate the percentage of participants who endorsed the new items (i.e., who marked response options “2” or “3”). Herein, credible participants from the *Neurotypical Control Group* and *ADHD Group* were combined into one sample. Items marked with an asterisk form the ADHD Credibility Index

As depicted in Fig. 1, twelve CAARS-ACI items were infrequently endorsed by the combined sample of credible controls and adults with ADHD (items 11, 14, 18, 24, 33, 35, 45, 49, 54, 58, 62, and 67). These items were equally divided between the four detection strategies upon which their development had been based: three items tapped supposed symptoms (items 11, 24, and 58), three items aimed to detect exaggerated complaints (items 18, 62, and 67), three items enquired about unusual symptom combinations (items 35, 49, and 54), and the remaining three items used selectivity of symptom reports (items 14, 33, and 45).

With twelve items having been selected, each of which was to be rated on a four-point scale, possible scores on the ADHD Credibility Index ranged from 0 to 36. Summary statistics for the ACI are presented in Table 2. Based on a collapsed sample of individuals from all groups (*N* = 1383), internal reliability of the new twelve-item index was high (Cronbach’s *α* = 0.94).

**Table 2:** Summary Statistics for ADHD Credibility Index (ACI) Scores by Group

	Neurotypical Control Group		ADHD Group		Simulation Group
	Credible	Overreporting	Credible	Non-Credible	
Median (MAD)	2 (2)	22 (5)	11 (4)	10.5 (5.5)	17 (7)
ACI-A	0 (0)	5 (1)	2 (1)	2.5 (1.5)	4 (2)
ACI-B	0 (0)	5.5 (1.5)	3 (1)	2 (1)	4 (2)
ACI-C	0 (0)	6 (1)	3 (1)	2 (1)	4 (2)
ACI-D	0 (0)	6 (1.5)	2 (1)	2 (2)	4 (2)
Range	25	23	32	17	36
Min—Max	0–25	7–30	0–32	2–19	0–36
Mode	0	24	5^a^	5	19
ACI-A	0	5	2	1	5
ACI-B	0	5.5	3	1	6
ACI-C	0	6	2	2	5
ACI-D	0	6	3	2	5

*MAD* median absolute deviation, *ACI-A* supposed symptoms subscale, *ACI-B* exaggerated symptoms subscale, *ACI-C* symptom combinations subscale, *ACI-D* selectivity subscale

^a^Multiple modes exist. The smallest value is shown here

As illustrated in Appendix 2 in ESM, 99.7% of credible controls (*n* = 997) and 94.7% of credible adults with ADHD (*n* = 90) produced a score at or below 21 on this index. No gender-specific differences in ACI were noted [*H*(2) = 1.870, *p* = 0.393]. However, results of a Kruskal–Wallis test showed statistically significant differences in ACI scores between age brackets [*H*(3) = 88.262, *p* < 0.01]. With the exception of 18- thru 29-year-olds, whose ACI scores did not differ significantly from 30- to 39-year-olds (*z* = 1.33, adjusted *p* = 1.00), post hoc tests revealed significant differences between all age groups (adjusted *p* < 0.01 in all cases).

Cut-off scores needed to ensure at least 90% specificity thus varied considerably between the groups. As shown in Table 3, a cut-off score of 5 was sufficient to ensure adequate specificity among controls aged 50 years or older. In contrast, a cut-off value of 21 was needed to guarantee comparable specificity among 30- thru 39-year-olds with ADHD. As sample sizes were very small for numerous age groups, we refrained from providing age-specific cut-off scores as part of the ADHD Credibility Index’ initial validation nonetheless. We examined a universal, conservative cut-off value instead. For all further analyses, sum scores above 21 were considered suspect.

**Table 3:** ADHD Credibility Index Scores needed to ensure at least 90% specificity

Age Group (years)	Group	*n*	Total		*n*	Male		*n*	Female	
			Cut-Off Score	% below		Cut-Off Score	% below		Cut-Off Score	% below
Total	Control + ADHD	1095	11	91.10	537	12	92.40	555	9	90.50
	Control Group	1000	8	91.70	493	9	90.70	504	7	91.50
	ADHD Group	95	21	94.70	44	21	95.50	51	20	90.20
18–29	Control + ADHD	141	13	91.50	53	16	92.50	87	12	92.00
	Control Group	109	12	92.70	36	15	91.70	72	8	93.10
	ADHD Group	32	17	93.80	17	17	94.10	15	17	93.30
30–39	Control + ADHD	221	16	90.00	92	17	91.30	129	14	90.70
	Control Group	191	12	90.60	80	16	90.00	111	8	91.00
	ADHD Group	30	21	93.30	12	21	100.0	18	23	94.40
40–49	Control + ADHD	244	11	90.20	116	12	93.10	127	10	90.60
	Control Group	228	9	90.80	111	11	91.00	116	6	90.50
	ADHD Group	16	18	93.80	5	13	100.0	12	18	90.90
50 +	Control + ADHD	487	6	91.80	274	6	91.20	212	6	92.50
	Control Group	470	5	91.90	264	5	91.70	205	5	92.20
	ADHD Group	17	27	94.10	10	27	100.0	7	22	100.0

Data shown here are based on credible participants’ responses only

### Association with symptoms of ADHD

As expected, patients with ADHD more commonly scored in the clinical range on the CAARS’ DSM scales than controls did (see Table 4). However, the percentage of symptomatic participants, who presented with suspect ACI scores, was consistently higher among controls than adults with ADHD. Whereas the percentage of patients with ADHD and suspect ACI scores ranged between 0% and approximately 7%, 22% of controls ‘symptomatic’ on the DSM Hyperactivity/Impulsivity (F) Scale scored above the ACI cut-off value. The percentage of participants without elevations on the DSM scales, whose ACI scores exceeded the cut-off value, lay below 1% in both groups.

**Table 4:** Association of ADHD symptomatology and ADHD Credibility Index (ACI) Scores

Scale	Group	Classification	%	ACI	
				% Not Suspect	% Suspect
CAARS DSM Inattention (E)	Control Group	No scale elevation	94.18	100.00	0.00
		Symptomatic	4.15	92.86	7.14
	ADHD Group	No scale elevation	9.47	100.00	0.00
		Symptomatic	43.16	100.00	0.00
	Total	No scale elevation	86.91	100.00	0.00
		Symptomatic	7.49	96.39	3.61
CAARS DSM Hyperactivity (F)	Control Group	No scale elevation	96.05	99.90	0.10
		Symptomatic	3.55	77.78	22.22
	ADHD Group	No scale elevation	56.84	100.00	0.00
		Symptomatic	32.63	93.55	6.45
	Total	No scale elevation	92.69	99.90	0.10
		Symptomatic	6.05	85.07	14.93
CAARS DSM Total (G)	Control Group	No scale elevation	95.16	100.00	0.00
		Symptomatic	3.65	91.89	8.11
	ADHD Group	No scale elevation	26.32	100.00	0.00
		Symptomatic	40.00	100.00	0.00
	Total	No scale elevation	89.26	100.00	0.00
		Symptomatic	6.77	96.00	4.00
CAARS ADHD Index (H)	Control Group	No scale elevation	97.24	99.90	0.10
		Symptomatic	2.47	64.00	36.00
	ADHD Group	No scale elevation	29.47	100.00	0.00
		Symptomatic	55.79	100.00	0.00
	Total	No scale elevation	91.43	99.90	0.10
		Symptomatic	7.04	88.46	11.54

Classifications are based on the CAARS DSM Scale *T*-Scores: ‘No Scale Elevation’ if *T* < 65, ‘Symptomatic’ if *T* ≥ 65. Percentages in the ‘%’ column do not add up to 100 as overreporting participants (*T* ≥ 80) are not reported here

### Utility of the ADHD Credibility Index in the detection of non-credible symptom report

We examined the ACI’s utility in discerning credible from non-credible self-report by comparing the *Credible ADHD Group* with the *Simulation Group* and the *Non-Credible ADHD Group* in a series of ROC analyses.

*Classification of non-credible symptom report in simulation design* Simulators’ scores were, on average, higher than ACI scores among genuine cases of adult ADHD (see Table 2), resulting in a small effect [*d* = 0.55, 95% CI (− 0.32, 1.41)]. Considering each subset of ACI items individually, the largest effect could be noted for inquiries into supposed symptoms. Items assessing exaggerated symptoms or selectivity of symptom reports yielded comparable results. The smallest effect emerged for the subscale tapping unusual symptom combinations. Effect sizes are summarized and illustrated in Appendix 3 in ESM.

**Fig. 2:**
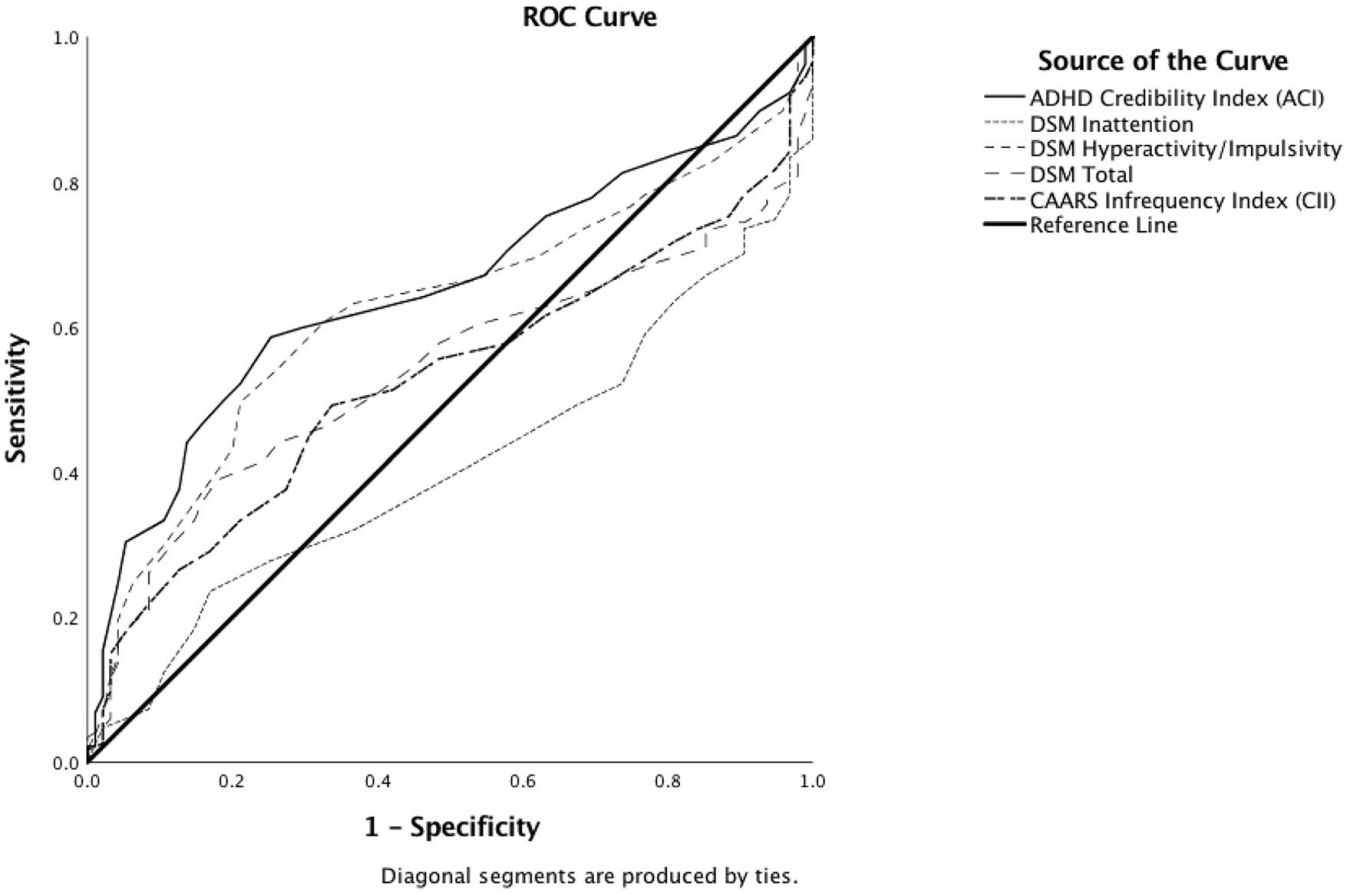
Receiver operating characteristics (ROC) curve indicating diagnostic accuracy of the ADHD Credibility Index (ACI), the CAARS DSM Scales, and the CAARS Infrequency Index (CII) in identifying feigned ADHD (Simulation Group, *n* = 234) relative to ADHD (ADHD Group, *n* = 95)

Applying a cut-off score of 21, the ACI correctly identified 71 simulators (30.34%) at a specificity of 98.50% (see Table 5). ROC analysis revealed an Area under the Curve of 0.651 [SE = 0.030, *p* < 0.01, 95% CI (0.591, 0.710)]. The ACI thereby outperformed the DSM Inattention (E) Scale and the DSM (G) Total in the detection of simulators, whereas the DSM Hyperactivity/Impulsivity (F) Scale yielded results comparable to those of the ACI (see Table 6 and Fig. 2). The CII correctly identified 112 simulators (46.28%). Specificity of the CII lay at 95.09%. Using this index as the criterion in a ROC Analysis resulted in an AUC of 0.527 [SE = 0.032, *p* = 0.44, 95% CI (0.465, 0.590)].

**Table 5:** Sensitivity, Specificity, Positive Predictive Value (PPV), and Negative Predictive Value (NPV) of the ADHD Credibility Index (ACI) and CAARS Infrequency Index (CII) in the Detection of Simulated ADHD, Non-Credible Adults with ADHD, and Overreport on CAARS DSM Scales

	Base Rate	Group		
		Simulation	Non-Credible ADHD	Overreport
ACI				
Sensitivity		30.34%	0.0%	38.34%
Specificity		98.50%	94.74%	98.81%
PPV	10	69.20%	0.0%	78.13%
	20	83.49%	0.0%	88.94%
	30	89.66%	0.0%	93.23%
	50	95.29%	0.0%	96.98%
NPV	10	92.71%	89.50%	93.52%
	20	84.98%	79.12%	86.50%
	30	76.74%	68.85%	78.90%
	50	58.58%	48.65%	61.58%
CII				
Sensitivity		46.28%	27.27%	64.53%
Specificity		95.09%	69.00%	96.86%
PPV	10	51.17%	8.90%	69.57%
	20	70.22%	18.03%	83.73%
	30	80.16%	27.38%	89.82%
	50	90.41%	46.80%	95.37%
NPV	10	94.09%	89.52%	96.09%
	20	87.62%	79.14%	91.61%
	30	80.51%	68.88%	86.44%
	50	63.90%	48.69%	73.20%

Participants were classified as Overreporters if their *T*-scores on any CAARS DSM Scale were ≧ 80

**Table 6:** Results of ROC analyses distinguishing credible adults with ADHD (*n* = 95) from simulators (*n* = 234)

	AUC	SE	*p*	95% CI	
				Lower	Upper
ACI	0.651	0.030	< 0.01*	0.591	0.710
CAARS DSM Inattention (E)	0.410	0.031	0.011*	0.349	0.472
CAARS DSM Hyperactivity/Impulsivity (F)	0.623	0.031	< 0.01*	0.561	0.684
CAARS DSM Total (G)	0.538	0.031	0.282	0.476	0.600
CII	0.527	0.032	0.435	0.465	0.590

*AUC* area under the curve, *ACI* ADHD Credibility Index, *CII* Conners’ Infrequency Index

*Statistically significant at *α* = 0.05

*Classification of non-credible patient report* Patients considered non-credible based on their TOMM or GET results presented with ADHD Credibility Index scores comparable to those of their credible counterparts (see Table 2). The effect size yielded by their comparison was negligible, irrespective of whether the complete index [*d* = 0.15, 95% CI (− 0.958, 1.240)] or its individual subscales were considered (see Appendix 3 in ESM). Indeed, no participant in this small subset of non-credible patients scored above the cut-off value on the ACI. Six non-credible adults with ADHD (27.27%) produced suspect scores on the CII. Specificity of the CII was 69.00% (see Table 5).

**Fig. 3:**
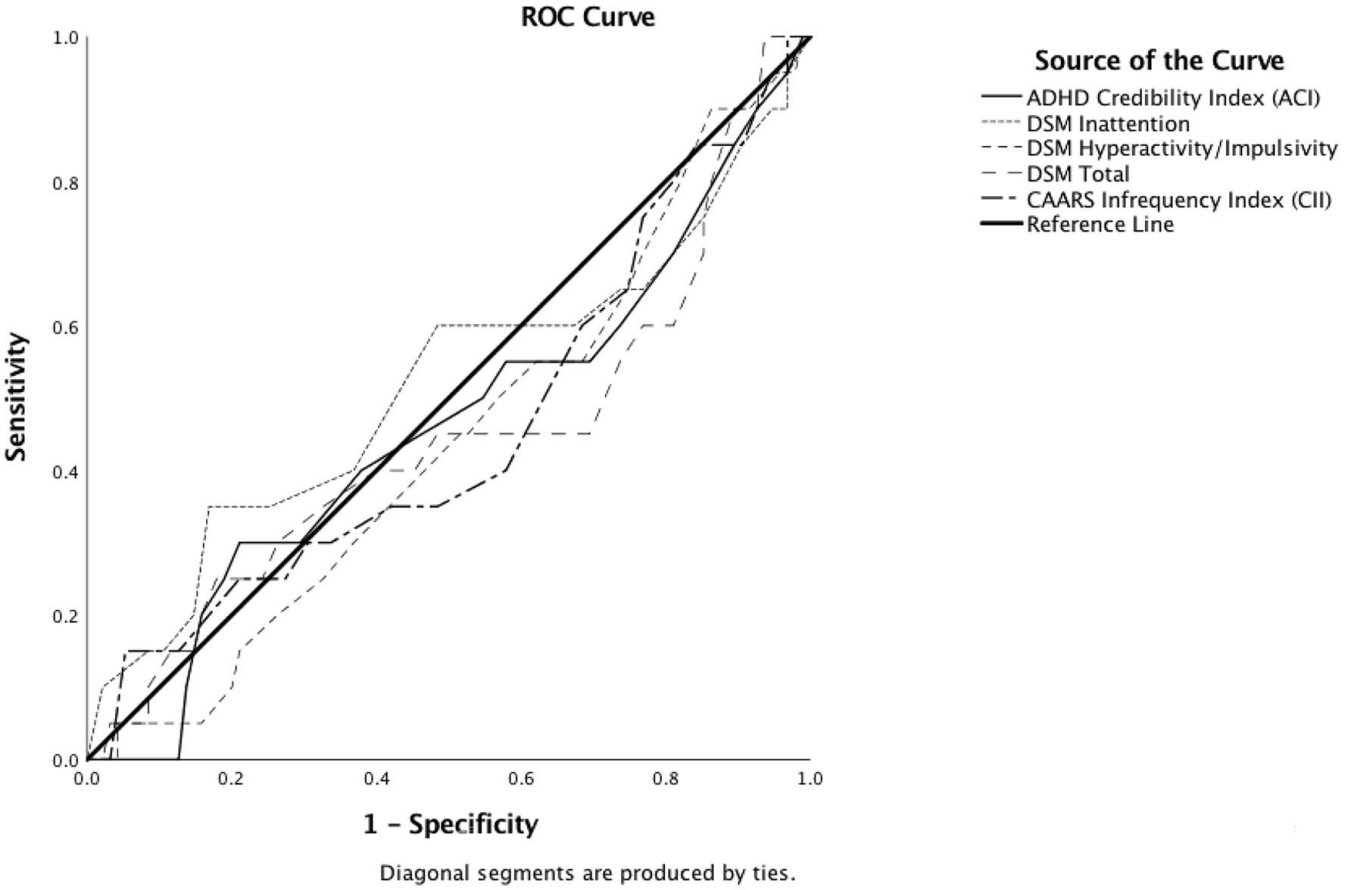
Receiver operating characteristics (ROC) curve illustrating diagnostic accuracy of the ADHD Credibility Index (ACI), the CAARS DSM Scales, and the CAARS Infrequency Index (CII) in discriminating non-credible adults with ADHD (non-credible ADHD Group, *n* = 20) from credible adults with ADHD (credible ADHD Group, *n* = 95)

ROC analyses showed that the diagnostic accuracy of the ACI, the CII, and the DSM Scales in discriminating credible from non-credible patients with ADHD did not differ significantly from chance (see Table 7 and Fig. 3).

**Table 7:** Results of ROC analyses distinguishing credible adults with ADHD (*n* = 95) from non-credible adults with ADHD (*n* = 20)

	AUC	SE	*p*	95% CI	
				Lower	Upper
ACI	0.462	0.075	0.598	0.315	0.609
CAARS DSM Inattention (E)	0.517	0.081	0.808	0.358	0.676
CAARS DSM Hyperactivity/Impulsivity (F)	0.442	0.069	0.417	0.307	0.577
CAARS DSM Total (G)	0.446	0.078	0.445	0.293	0.598
CII	0.457	0.075	0.548	0.311	0.603

*AUC* area under the curve, *ACI* ADHD Credibility Index, *CII* Conners’ Infrequency Index

### Comparison with existing validity indicators

**Fig. 4:**
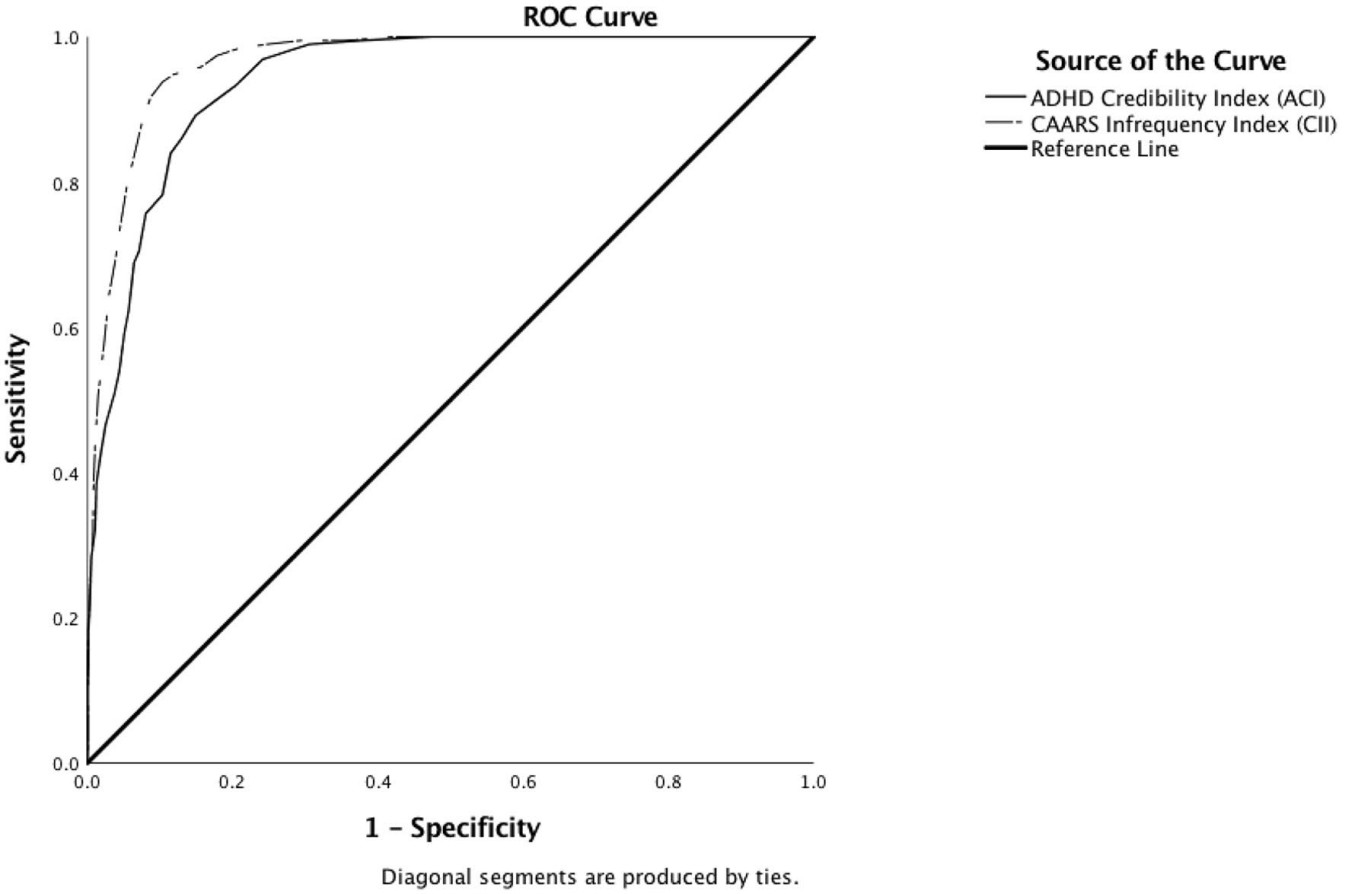
Receiver operating characteristics (ROC) curve indicating diagnostic accuracy of the ADHD Credibility Index (ACI) and the CAARS Infrequency Index (CII) in distinguishing over-reporters (i.e., participants from any group who presented with T-Scores ≥ 80 on any DSM Sale, *n* = 193) from participants who scored in the unremarkable range on all DSM Scales (*n* = 1174)

*Classification of over-reported symptoms on DSM scales* Like Suhr et al. (2011), we investigated the ADHD Credibility Index’ ability to discern unremarkable response patterns from over-reporting (i.e., *T*-Scores > 80). To this end, we collapsed all groups into one and split the combined sample into credible and over-reporting participants. In a first analysis, participants were considered over-reporters if their *T*-Score on *any* DSM Scale exceeded 80. Using the ACI as the metric predicting over-report, ROC analysis showed an AUC of 0.941 [SE = 0.007, *p* < 0.01, 95% CI (0.928, 0.954)]. Repeating the same analysis for over-report on individual DSM scales showed comparable results for the detection of over-report on the DSM Hyperactivity/Impulsivity (F) Scale and the DSM Total (G). The smallest AUC could be noted for the DSM Inattention (E) Scale (see Table 8 and Fig. 4*)*. Suhr’s Infrequency Index (CII) outperformed the ACI in the classification of over-report. Using the CII to detect over-report on any given DSM scale resulted in an AUC of 0.966 [SE = 0.005, *p* < 0.01, 95% CI (0.957, 0.975)]. Sensitivity and specificity of the ACI and CII in detecting over-report on any DSM scale are presented in Table 5.

**Table 8:** Results of ROC analyses distinguishing participants with unremarkable *T*-scores on the DSM scales (*n* = 1174) from over-reporters (*n* = 193)

	Overreport on	AUC	SE	*p*	95%-*CI*	
					Lower	Upper
ACI	Any CAARS DSM Scale	0.941	0.007	< 0.01*	0.928	0.954
	CAARS DSM Inattention (E)	0.928	0.008	< 0.01*	0.913	0.944
	CAARS DSM Hyperactivity (F)	0.959	0.007	< 0.01*	0.946	0.972
	CAARS DSM Total (G)	0.952	0.006	< 0.01*	0.940	0.964
CII	Any CAARS DSM Scale	0.966	0.005	< 0.01*	0.957	0.975
	CAARS DSM Inattention (E)	0.958	0.006	< 0.01*	0.947	0.969
	CAARS DSM Hyperactivity (F)	0.978	0.004	< 0.01*	0.971	0.986
	CAARS DSM Total (G)	0.970	0.004	< 0.01*	0.962	0.979

*AUC* area under the curve, *ACI* ADHD Credibility Index, *CII* Conners’ Infrequency Index

*Statistically significant at *α* = 0.05

*Agreement with existing CAARS validity indicators* To examine the agreement of established validity indicators and the new infrequency index, we contrasted classifications based on *T*-Scores exceeding 80 (as recommended in the CAARS manual) (Conners et al. 1999), Inconsistency Indices equal to or above 8, and CII scores equal to or above 21 with those of the ADHD Credibility Index. Herein, we considered all groups, including over-reporting controls and non-credible patients with ADHD.

While the percentage of participants showing *T-*Score elevations in the suspect range varied markedly depending on which DSM scale was considered, such elevations were generally most common among simulators, followed by credible and non-credible adults with ADHD, and lastly controls (see Table 9). Over-report on the DSM scales and suspect ACI results more commonly co-occurred for simulators and controls than credible patients with ADHD. Indeed, the higher the percentage of participants in the credible ADHD Group, whose *T*-Scores fell into the suspect range, the lower the percentage among them who produced suspect ACI results.

**Table 9:** Agreement between ADHD Credibility Index and Overreport on CAARS DSM Scales

Scale	Group	%	ADHD Credibility Index	
			Not Suspect (%)	Suspect (%)
CAARS DSM Inattention (E)	Control Group	1.68	47.06	52.94
	ADHD Group	47.37	88.89	11.11
	Simulation Group	42.74	47.00	53.00
	Non-Credible ADHD Group	50.00	100.00	0.00
	Total	12.63	61.05	38.95
CAARS DSM Hyperactivity/Impulsivity (F)	Control Group	0.39	25.00	75.00
	ADHD Group	10.53	70.00	30.00
	Simulation Group	32.05	38.67	61.33
	Non-Credible ADHD Group	5.00	100.00	0.00
	Total	6.61	42.22	57.78
CAARS DSM Total (G)	Control Group	1.18	25.00	75.00
	ADHD Group	33.68	84.38	15.62
	Simulation Group	47.44	46.85	53.15
	Non-Credible ADHD Group	35.00	100.00	0.00
	Total	11.89	54.94	45.06
CAARS ADHD Index (H)	Control Group	0.30	33.33	66.67
	ADHD Group	14.74	64.29	35.71
	Simulation Group	8.12	0.00	100.00
	Non-Credible ADHD Group	5.00	100.00	0.00
	Total	2.72	29.73	70.27

Column denoted ‘%’ shows the percentage of participants within the respective group, whose *T*-Scores fell into the suspect range (i.e., *T* ≥ 80)

Collapsing all groups into one, 8.57% of participants responded in an inconsistent manner (see Table 10). Approximately 11% of these respondents produced suspect scores on the ACI. The highest agreement between the ACI and the CAARS Inconsistency Index could be noted for the *Simulation Group,* 29.27% of whom were identified by the ACI.

**Table 10:** Agreement between ADHD Credibility Index and Existing Validity Indicators

Index	Group	Classification	%	ADHD Credibility Index	
				% Not Suspect	% Suspect
Inconsistency Index	Control Group	Not Inconsistent	94.89	98.86	1.14
		Inconsistent	5.11	98.08	1.92
	ADHD Group	Not Inconsistent	76.60	93.06	6.94
		Inconsistent	23.40	100.00	0.00
	Simulation Group	Not Inconsistent	82.48	69.43	30.57
		Inconsistent	17.52	70.73	29.27
	Non-Credible ADHD Group	Not Inconsistent	90.00	100.00	0.00
		Inconsistent	10.00	100.00	0.00
	Total	Not Inconsistent	91.43	94.00	6.00
		Inconsistent	8.57	88.89	11.11
CII	Control Group	Not Suspect	98.23	99.80	0.20
		Suspect	1.77	44.44	55.56
	ADHD Group	Not Suspect	69.47	100.00	0.00
		Suspect	30.53	82.76	17.24
	Simulation Group	Not Suspect	55.13	94.57	5.43
		Suspect	44.87	39.05	60.95
	Non-Credible ADHD Group	Not Suspect	70.00	100.00	0.00
		Suspect	30.00	100.00	0.00
	Total	Not Suspect	88.44	99.26	0.74
		Suspect	11.56	50.00	50.00

Suspect results on the CAARS Infrequency Index were more common than inconsistent responding, with 11.56% of all participants presenting with scores above 20. The ACI identified 50% of these participants. Agreement between the ACI and the CII was highest among simulators (60.95%) and over-reporting controls (55.56%) (see Table 10).

## Discussion

The current study described the development and initial validation of a new disorder-specific infrequency index aiding the detection of non-credible adult ADHD, the ADHD Credibility Index (ACI). Once evaluated for infrequency among credible adults with ADHD and their neurotypical counterparts, twelve of fifteen newly written items remained and were summed to form the ACI. Four subscales, all corresponding to the detection strategies which informed the development of ACI items, were composed of three items each: supposed symptoms, exaggerated symptoms, symptom combinations, and selectivity of symptom report.

Utility of the ADHD Credibility Index (ACI) in the detection of non-credible, self-reported symptoms of ADHD was dependent on the sample under study. The ACI detected instructed simulators at rates comparable to existing embedded validity indicators, particularly those described in early studies on the CII (Suhr et al. 2011; Walls et al. 2017). The CII showed greater sensitivity to feigned ADHD than did the ACI, while retaining an only marginally lower specificity. ROC analyses, on the other hand, suggested the ACI’s overall classification accuracy to be superior to that of the CII. Neither of the indices classified simulators at consistently higher rates than the CAARS DSM scales, though: the DSM Hyperactivity/Impulsivity (F) Scale was comparable to the ACI in identifying instructed simulators, and the DSM Total (G) Scale yielded results akin to those of the CII. Solely the DSM Inattention (E) was less accurate in the detection of the Simulation Group than either infrequency index.

Neither the ACI nor the CII showed satisfactory classification accuracy when used to identify adults with ADHD who had failed an independent performance validity test (PVT). ROC analysis indicated that neither of these infrequency indices, nor the CAARS DSM scales, performed significantly above chance. In light of the non-credible group’s small sample size, these results ought to be interpreted with utmost caution. Yet, they may underscore divergence of results provided by SVTs and PVTs (Copeland et al. 2016; Hirsch and Christiansen 2018; Larrabee 2012; Van Dyke et al. 2013; White et al. 2020).

In contrast, both ACI and CII were useful in detecting symptom over-report on the three DSM scales included in the CAARS, which the authors propose to be indicative of severe symptomatology or non-credible responding (Conners et al. 1999). Despite an association between ADHD symptomatology and ACI scores, the new infrequency index identified a smaller subset of over-reporting patients than neurotypical individuals whose *T*-Scores fell in the suspect range. Whether this effect was due to genuine patients with particularly pronounced symptoms being classified as credible by the ACI—as would be desirable—remains unverified as of yet.

Results do provide preliminary evidence of the ACI identifying a different subgroup of respondents than the CII (see Table 11). The two infrequency indices agreed in approximately 94% of cases. Depending on which group was considered, concordance ranged from 99% for the control group to 75% for credible and 70% for non-credible adults with ADHD, respectively. Agreement in the *Overreporting Control* (89%) and *Simulation* (79%) *Groups* fell in between. Divergence most commonly resulted from respondents being identified as suspect by the CII but not the ACI, which detected 50% of participants whose CII scores fell above the cut-off value. Approximately half of those *not* identified by the ACI were simulators, illustrating the CII’s superior sensitivity to feigned instances of ADHD. However, 30% of respondents identified only by the CII were credible patients with ADHD and 11% were controls. Seeing that CII items stem from an instrument intended to measure symptoms of ADHD, this greater number of adults with the disorder being identified by the CII—compared to the ACI—may come to no surprise. Minor overlap between the CII and ACI is in line with findings suggesting that the CII and EI, too, each detect different subgroups of examinees (Harrison et al. 2019). As expected, agreement between the infrequency indices and the CAARS’ inconsistency index was low.

**Table 11:** Agreement between ADHD Credibility Index (ACI) and CAARS Infrequency Index (CII)

ACI suspect?	CII suspect?		
	No	Yes	
No	1200	79	1279
Yes	9	79	88
	1209	158	

Considering individual subscales rather than the ACI sum score, differences emerged between the four detection strategies which formed the ACI’s theoretical basis. Inquiries into supposed symptoms revealed a medium effect (*d* = 0.75) and therefore the largest difference between genuine cases of adult ADHD and simulators. Since items of this subscale tap complaints laypeople may erroneously associate with ADHD, this is consistent with previous studies recommending the use of stereotypes and misconceptions in the detection of feigned ADHD (Courrégé et al. 2019; Robinson and Rogers 2018). Another detection strategy, which has been considered promising due to the ease with which it can be adapted to specific disorders, is based on the combination of symptoms rarely reported as co-occurring by genuine patients. Rogers introduced such items as part of the Structured Interview of Reported Symptoms (Rogers et al. 2010). While the SIRS has been developed to assist the detection of feigned psychiatric complaints, rather than neurodevelopmental disorders, such as ADHD, its Symptom Combinations subscale showed some utility in the detection of simulated ADHD (Becke et al. 2019). ACI items which assessed ADHD-specific adaptations of this strategy, however, yielded the smallest effect of all subscales (*d* = 0.22). This was due to a substantial number of participants with ADHD endorsing these items, suggesting that the presented combinations of symptoms were not sufficiently rare in our sample of genuine patients after all. Subscales examining exaggerated symptoms and selectivity of symptom complaints revealed small effects for the comparison of credible patients with ADHD and instructed simulators. Interpretation of these effect sizes requires caution, as data were non-normally distributed.

### Limitations

Several limitations inherent in the present study may inform future research. As a consequence of differences in recruitment procedures, groups differed significantly on demographic variables, such as age and gender. Simulators were recruited from a population highly pertinent to research on simulated ADHD: university students. However, they were significantly younger than participants in other groups, which is particularly relevant in light of age-related differences in ACI scores. Similarly, gender distributions were unequal between simulators and the remaining groups. Certain subgroups of participants, such as female patients with ADHD aged 50 years or older, were very small. We therefore decided not to provide gender- or age-specific cut-off scores, even though results suggest they may increase the ACI’s classification accuracy.

While we compared the ACI to the CII, juxtaposition of the ACI and EI was impossible as our experimental version of the CAARS did not include the additional items constituting the EI. The ACI’s classification accuracy may therefore only be compared to data presented in Harrison and Armstrong’s (2016) original study. Classification accuracy of the new ACI was on par with the low end of sensitivity reported for the EI, which ranged from 24 to 69%. Specificity of the ACI was at least comparable to that of the EI, if not marginally superior.

The current study did not include a clinical control group or a group of simulators instructed to feign general psychological pathology rather than ADHD. Inclusion of the former could provide additional information on the association between ACI scores and general psychological distress or symptomatology and thus assist in ensuring low false positive error rates. Discerning overall symptom over-report or ‘faking bad’ from instances of feigned ADHD may be fostered by including a group of simulators instructed to feign psychopathology in a broad sense, rather than ADHD specifically.

## Concluding remarks

While less sensitive to instances of feigned ADHD than the CII and recently introduced measures, such as the INF Scale developed by Courrégé et al. (2019) or the Ds-ADHD (Robinson and Rogers 2018), the ACI may be a useful adjunct measure in the assessment of credibility of self-reported ADHD. Its classification accuracy, as determined in ROC analyses, was on par with existing validity indicators, yet initial data suggest it identified a different subset of respondents than the CII. Application of a universal, conservative cut-off score may have stymied the identification of simulators and non-credible adults with ADHD, but has ensured excellent specificity. The ACI proved most useful in discerning symptom over-report from unremarkable response patterns. This underscores Robinson’s and Rogers’ (2018) call to aim for the detection of different feigning presentations, such as distinguishing feigned ADHD from unspecific feigned psychopathology, rather than relying on rare symptoms and the detection of symptom over-endorsement. Cross-validation, including the evaluation of refined cut scores, could help to further elucidate the instrument’s utility in distinguishing specific forms of feigning from such general over-report. Increasing its classification accuracy may call for the integration of multiple variables, as illustrated by other promising approaches to uncovering feigned ADHD (see, for example, Aita et al. 2017; Fuermaier et al. 2016a, b). Such a multivariate approach could combine ACI items with individual CII items or elevated scale scores, as demonstrated by Harrison and Armstrong (2016) in the development of the EI. Erdodi (2019) detailed how joining several data points makes the “internal logic [of validity tests] impenetrable to examinee[s]”, thus lowering the instruments’ vulnerability to coaching and making it increasingly harder for respondents to influence the test results in their favor.

## Supplementary Information

Below is the link to the electronic supplementary material.

Supplementary file1 (DOCX 482 KB)

## Data Availability

The data that support the findings of this study are available from the corresponding author, MB, upon reasonable request.
