# Correction to: Non-credible symptom report in the clinical evaluation of adult ADHD: development and initial validation of a new validity index embedded in the Conners’ adult ADHD rating scales

**DOI:** 10.1007/s00702-022-02533-1

**Published:** 2022-08-17

**Authors:** Miriam Becke, Lara Tucha, Matthias Weisbrod, Steffen Aschenbrenner, Oliver Tucha, Anselm B. M. Fuermaier

**Affiliations:** 1 Department of Clinical and Developmental Neuropsychology, Faculty of Behavioural and Social Sciences, University of Groningen, Grote Kruisstraat 2/1, 9712 TS Groningen, The Netherlands; 2 Department of Psychiatry and Psychotherapy, University Medical Center Rostock, Gehlsheimer Str. 20, 18147 Rostock, Germany; 3 Department of Psychiatry and Psychotherapy, SRH Clinic Karlsbad-Langensteinbach, 76307 Karlsbad, Germany; 4 Department of General Psychiatry, Center of Psychosocial Medicine, University of Heidelberg, 69115 Heidelberg, Germany; 5 Department of Clinical Psychology and Neuropsychology, SRH Clinic Karlsbad-Langensteinbach, 76307 Karlsbad, Germany

## Correction to: Journal of Neural Transmission (2021) 128:1045–1063 10.1007/s00702-021-02318-y

Due to an administrative error, 73 individuals were wrongfully assigned to the *Simulation Group*. Correction of this error consequently reduced its sample size from 242 to 169 individuals.

This did not affect the development of the ADHD Credibility Index (ACI). However, it resulted in an underestimation of the index’ classification accuracy in its initial validation.

The corrected demographic data of the *Simulation Group* (see amended Table 1) differed from those of the other experimental groups as described in the original publication. Participants of this group were still significantly younger than participants in the *ADHD Groups* (credible: *z* = 7.357, adjusted *p* < 0.01; non-credible: *z* = 3.819, adjusted *p* < 0.01) and *Control Groups* (credible: *z* = 20.681, adjusted *p* < 0.01; overreporting: *z* = 3.557, adjusted *p* < 0.01). The gender distribution in this group also differed from the *Credible* (*χ*^2^ (1) = 42.518, *p* < 0.01) and *Overreporting Control Groups* (*χ*^2^ (1) = 20.289, *p* < 0.01) as well as the *ADHD Groups* (credible: *χ*^2^ (1) = 16.176, *p* < 0.01; non-credible: *χ*^2^ (1) = 13.327, *p* = 0.01). In terms of education, instructed simulators differed from credible participants in the *Control Group* (*z* = − 7.611, adjusted *p* < 0.01) and the *ADHD Group* (*z* =  − 3.660, adjusted *p* < 0.01), but not from overreporting controls (*z* = 1.864, adjusted *p* = 0.623) or non-credible patients with ADHD (*z* = 0.014, adjusted *p* = 1.00).

**Table 1.** Descriptive data by group

	Neurotypical control group (*n* = 1019)		ADHD group (*n* = 122)		Simulation group (*n* = 169)
	Credible (*n* = 1001)	Overreporting (*n* = 18)	Credible (*n* = 100)	Non-credible (*n* = 22)	
Age (years)					
Median (MAD)	49 (11)	32 (4)	34 (9)	31.50 (10.5)	20 (1)
Range	40	33	62	42	41
Sex (m/f)	494/504	13/5	46/54	13/9	38/131
%	49.4/50.3*	72.2/27.8	46.0/54.0	59.1/40.9	22.5/77.5
Education					
Years					
Median (MAD)	13 (3)	13 (3)	13 (3)	14 (2)	13 (1)
Range	10	10	16	15	14
ADHD Symptomatology					
Past^a^					
Median (MAD)			40.0 (10)	40.5 (14.5)	13.0 (6)
Range			70.0	53.5	48.0
Present^b^					
Median (MAD)			31.0 (6)	28.5 (5.5)	10.0 (4)
Range			53.0	40.0	45.0

*MAD* median absolute deviation

*Three participants did not disclose their gender

^a^Wender Utah Rating Scale

^b^ADHD Self-Report Scale

Correcting the *Simulation Group* further required the revision of Tables 2, 5, 6, 8, 9, 10, 11. With the exception of the following findings, the pattern of results remained unchanged. The validity indicators under study showed overall **higher** sensitivity rates and **larger** effect sizes than previously reported. Rather than the small effect described in the original publication, the ACI yielded a large effect for the comparison of instructed simulators and credible adults with ADHD (*d* = 1.29, 95% CI [0.49, 2.09]). As was previously the case, the largest effect could be observed on the Supposed Symptoms subscale, followed by Exaggerated Symptoms, Selectivity, and lastly Symptom Combinations (see amended Appendix 3). Additionally, changes in classification accuracy were noted for two DSM scales (see amended Table 6). While previously significant, ROC analysis showed a statistically non-significant result for the DSM Inattention (E) scale. In contrast, the DSM Total (G), yielded a statistically significant result upon correction. These changes did not affect the conclusions drawn from the results.

**Table 2.** Summary statistics for ADHD Credibility Index (ACI) scores by group

	Neurotypical control group		ADHD group		Simulation group
	Credible	Overreporting	Credible	Non-credible	
Median (MAD)	2 (2)	22 (5)	11 (4)	10.5 (5.5)	20 (4)
ACI-A	0 (0)	5 (1)	2 (1)	2.5 (1.5)	5 (1)
ACI-B	0 (0)	5.5 (1.5)	3 (1)	2 (1)	5 (1)
ACI-C	0 (0)	6 (1)	3 (1)	2 (1)	5 (1)
ACI-D	0 (0)	6 (1.5)	2 (1)	2 (2)	5 (1)
Range	25	23	32	17	35
Min–max	0–25	7–30	0–32	2–19	1–36
Mode	0	24	5^a^	5	19
ACI-A	0	5	2	1	5
ACI-B	0	5.5	3	1	6
ACI-C	0	6	2	2	5
ACI-D	0	6	3	2	5

*MAD* Median Absolute Deviation, *ACI-A* Supposed Symptoms Subscale, *ACI-B* Exaggerated Symptoms Subscale, *ACI-C* Symptom Combinations Subscale, *ACI-D* Selectivity Subscale

^a^Multiple modes exist. The smallest value is shown here.

**Table 5.** Sensitivity, specificity, positive predictive value (PPV), and negative predictive value (NPV) of the ADHD Credibility Index (ACI) and CAARS Infrequency Index (CII) in the detection of simulated ADHD, non-credible adults with ADHD, and overreport on CAARS DSM Scales

	Base rate	Group		
		Simulation	Non-credible ADHD	Overreport
ACI				
Sensitivity		44.10%	0.0%	38.74%
Specificity		94.74%	94.74%	98.73%
PPV	10	48.21%	0.0%	77.23%
	20	67.69%	0.0%	88.41%
	30	78.22%	0.0%	92.90%
	50	89.34%	0.0%	96.83%
NPV	10	93.85%	89.50%	93.55%
	20	87.14%	79.12%	86.57%
	30	79.82%	68.85%	78.99%
	50	62.89%	48.65%	61.71%
CII				
Sensitivity		65.09%	27.27%	65.17%
Specificity		69.00%	69.00%	96.84%
PPV	10	18.92%	8.90%	69.65%
	20	34.42%	18.03%	83.77%
	30	47.36%	27.38%	89.85%
	50	67.74%	46.80%	95.38%
NPV	10	94.68%	89.52%	96.16%
	20	88.77%	79.14%	91.75%
	30	82.18%	68.88%	86.65%
	50	66.40%	48.69%	73.55%

Participants were classified as Overreporters if their *T*-Scores on any CAARS DSM Scale were ≧ 80

**Table 6.** Results of ROC analyses distinguishing credible adults with ADHD (*n* = 95) from simulators (*n* = 161)

	AUC	SE	*p*	95% CI	
				Lower	Upper
ACI	0.825	0.027	< 0.01*	0.773	0.878
DSM inattention (E)	0.565	0.037	0.084	0.493	0.636
DSM hyperactivity/impulsivity (F)	0.820	0.028	< 0.01*	0.764	0.875
DSM total (G)	0.743	0.032	< 0.01*	0.681	0.805
CII	0.716	0.033	< 0.01*	0.652	0.780

*AUC *area under the curve, *ACI* ADHD Credibility Index, *CII* Conners’ Infrequency Index

*Statistically significant at *α* = 0.05

**Table 8.** Results of ROC analyses distinguishing participants with unremarkable *T*-Scores on the DSM Scales (*n* = 1103) from Overreporters (*n* = 191)

	Overreport on	AUC	SE	*p*	95% CI	
					Lower	Upper
ACI	Any DSM Scale	0.944	0.006	< 0.01*	0.932	0.957
	DSM inattention (E)	0.931	0.007	< 0.01*	0.917	0.946
	DSM hyperactivity (F)	0.959	0.006	< 0.01*	0.947	0.972
	DSM total (G)	0.954	0.006	< 0.01*	0.943	0.966
CII	Any DSM Scale	0.966	0.005	< 0.01*	0.957	0.976
	DSM inattention (E)	0.958	0.006	< 0.01*	0.947	0.969
	DSM hyperactivity (F)	0.978	0.004	< 0.01*	0.970	0.985
	DSM total (G)	0.970	0.004	< 0.01*	0.962	0.979

*AUC* Area under the Curve, *ACI* ADHD Credibility Index, *CII* Conners’ Infrequency Index

*Statistically significant at α = 0.05

**Table 9.** Agreement between ADHD Credibility Index and overreport on DSM scales

Scale	Group	%	ADHD Credibility Index	
			Not suspect (%)	Suspect (%)
DSM inattention (E)	Control group	1.68	47.06	52.94
	ADHD group	47.37	88.89	11.11
	Simulation group	60.87	45.92	54.08
	Non-credible ADHD group	50.00	100.00	0.00
	Total	13.19	60.59	39.41
DSM hyperactivity/impulsivity (F)	Control group	0.39	25.00	75.00
	ADHD group	10.53	70.00	30.00
	Simulation group	45.96	37.84	62.16
	Non-credible ADHD group	5.00	100.00	0.00
	Total	6.90	41.57	58.43
DSM total (G)	Control group	1.18	25.00	75.00
	ADHD group	33.68	84.38	15.62
	Simulation group	67.70	45.87	54.13
	Non-credible ADHD group	35.00	100.00	0.00
	Total	12.41	54.38	45.62
ADHD Index (H)	Control group	0.30	33.33	66.67
	ADHD group	14.74	64.29	35.71
	Simulation group	11.80	0.00	100.00
	Non-credible ADHD group	5.00	100.00	0.00
	Total	2.87	29.73	70.27

Column denoted ‘%’ shows the percentage of participants within the respective group, whose *T*-Scores fell into the suspect range (i.e. *T *≥ 80)

**Table 10.** Agreement between ADHD Credibility Index and existing validity indicators

Index	Group	Classification	%	ACI	
				% Not Suspect	% Suspect
Inconsistency Index	Control group	Not inconsistent	94.89	98.86	1.14
		Inconsistent	5.11	98.08	1.92
	ADHD group	Not inconsistent	76.60	93.06	6.94
		Inconsistent	23.40	100.00	0.00
	Simulation group	Not inconsistent	80.75	54.62	45.38
		Inconsistent	19.25	61.29	38.71
	Non-credible ADHD group	Not inconsistent	90.00	100.00	0.00
		Inconsistent	10.00	100.00	0.00
	Total	Not inconsistent	91.72	93.68	6.32
		Inconsistent	8.28	87.85	12.15
CII	Control group	Not suspect	98.23	99.80	0.20
		Suspect	1.77	44.44	55.56
	ADHD group	Not suspect	69.47	100.00	0.00
		Suspect	30.53	82.76	17.24
	Simulation group	Not suspect	36.02	87.93	12.07
		Suspect	63.98	37.86	62.14
	Non-credible ADHD group	Not suspect	70.00	100.00	0.00
		Suspect	30.00	100.00	0.00
	Total	Not suspect	87.94	99.21	0.79
		Suspect	12.06	49.36	50.64

**Table 11.** Agreement between ADHD Credibility Index (ACI) and CAARS Infrequency Index (CII)

ACI suspect?	CII suspect?		
	No	Yes	
No	1129	77	1206
Yes	9	79	88
	1138	156	

## Appendix 3. Effect Sizes (d) with 95%-Confidence Intervals

**Table:**

	Credible ADHD group (*n* = 100) vs. simulation group (*n* = 169)			Credible ADHD group (*n* = 100) vs. non-credible ADHD group (*n* = 22)		
	*d*	Lower	Upper	*d*	Lower	Upper
ACI	1.292	0.486	2.098	0.146	− 0.958	1.249
ACI-A	1.478	1.256	1.700	0.006	− 0.297	0.309
ACI-B	1.203	0.970	1.436	0.306	− 0.056	0.667
ACI-C	0.736	0.480	0.993	0.164	− 0.207	0.535
ACI-D	0.994	0.761	1.227	0.073	− 0.273	0.419

*ACI* ADHD Credibility Index, *ACI-A* Supposed Symptoms Subscale, *ACI-B* Exaggerated Symptoms Subscale, *ACI-C* Symptom Combinations Subscale, *ACI-D* Selectivity Subscale

