# Supplementary material for: Non-credible symptom report in the clinical evaluation of adult ADHD: development and initial validation of a new validity index embedded in the Conners’ adult ADHD rating scales
Source: J Neural Transm (Vienna). 2021 Mar 2;128(7):1045–63. doi: 10.1007/s00702-021-02318-y (PMC8295107; doi:10.1007/s00702-021-02318-y)
Supplement: Supplementary file 1 — Supplementary file1 (DOCX 482 KB) [file 702_2021_2318_MOESM1_ESM.docx]

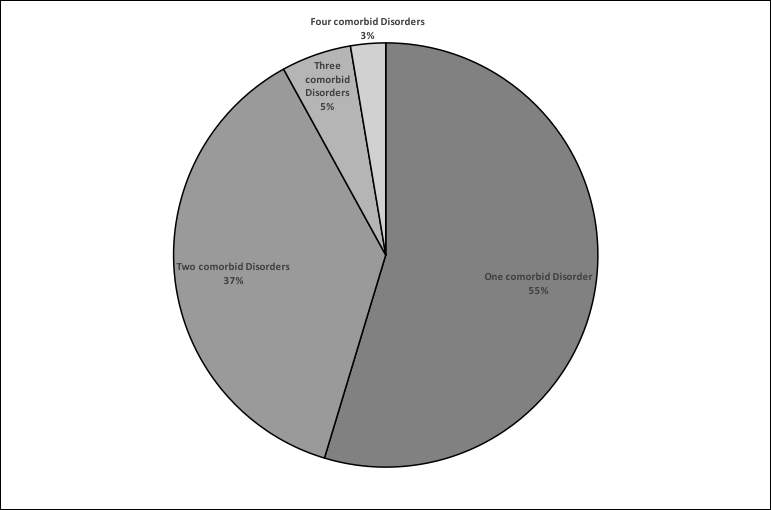


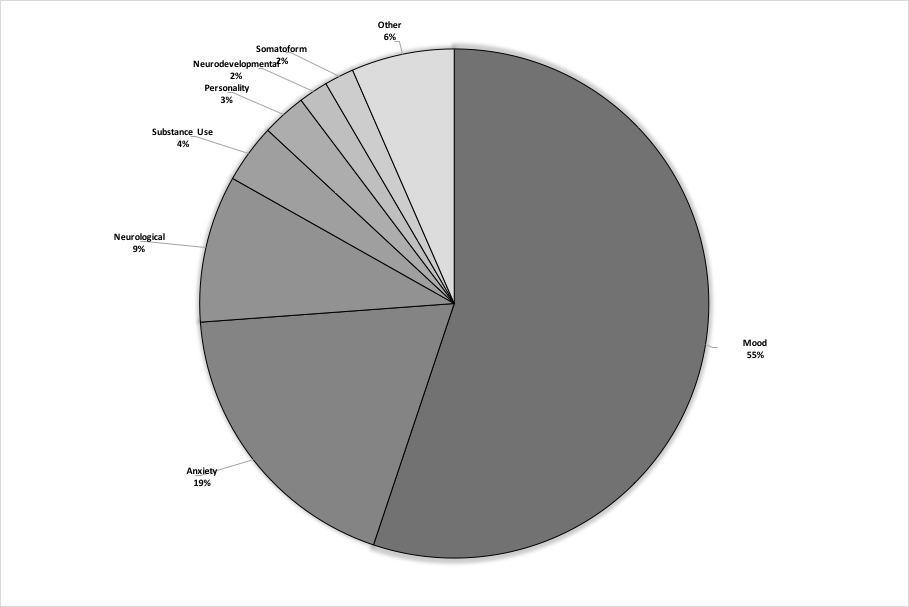


*Appendix 1. Psychiatric and Neurological Comorbidities in ADHD Group.* Top half illustrates the number of comorbidities reported by credible (n = 100) and non-credible (n = 22) patients who had received additional diagnoses alongside ADHD. Lower half shows which percentage of these diagnoses fell into various diagnostic clusters.


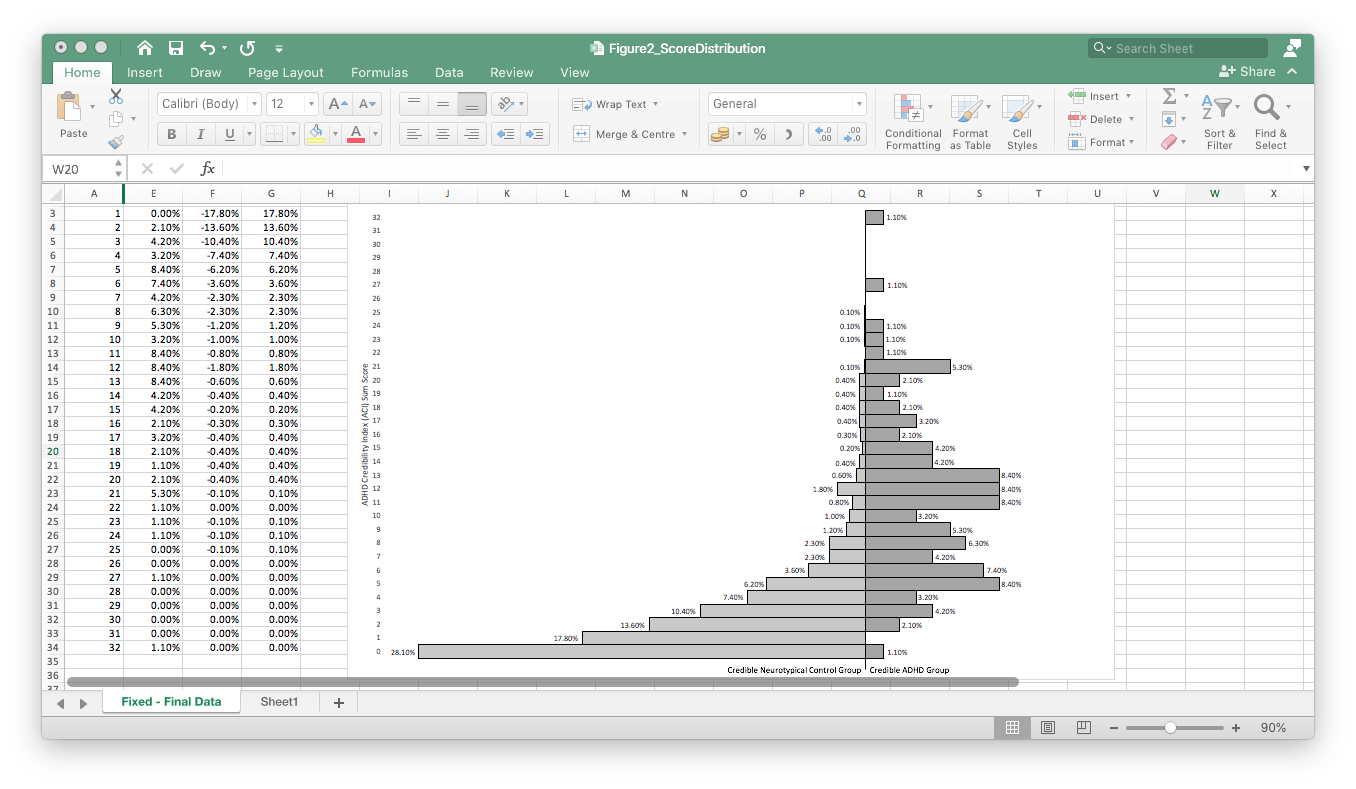


*Appendix 2*. Distribution of Sum Scores on the ADHD Credibility Index (ACI). Data depicted here include credible participants

*Appendix 3.* Effect Sizes (*d*) Yielded by the Comparison of Patients with ADHD (n = 100) and Simulators (n = 234).


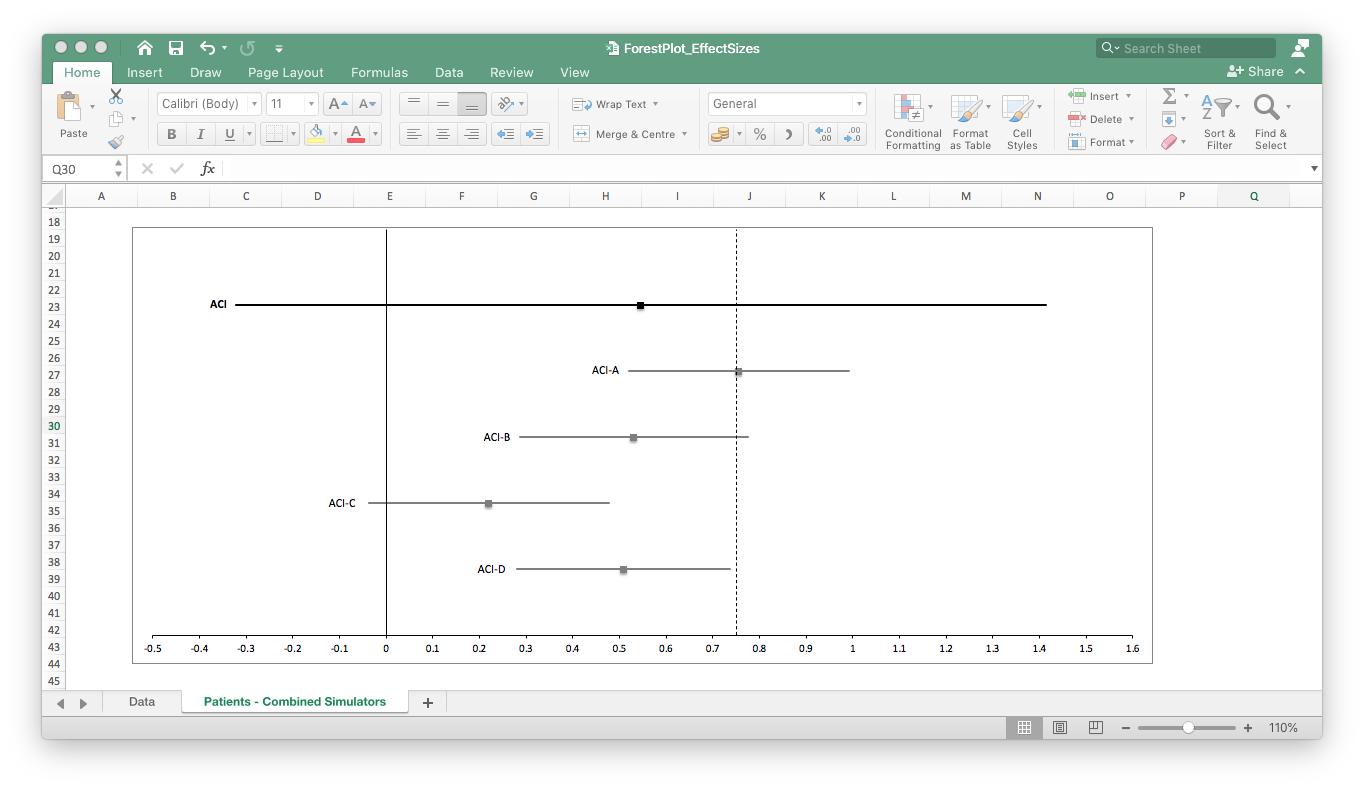


*Notes.* ACI = ADHD Credibility Index; ACI-A = Supposed Symptoms Subscale; ACI-B = Exaggerated Symptoms Subscale; ACI-C = Symptom Combinations Subscale; ACI-D = Selectivity Subscale; dashed line indicates a medium effect size of *d* = 0.75 (Rogers & Bender, 2018).

*Appendix 3.* Effect Sizes (*d*) with 95%-Confidence Intervals.

|  | | Credible ADHD Group (n = 100) vs. Simulation Group (n = 234) | | |  | Credible ADHD Group (n = 100) vs.  Non-Credible ADHD Group (n =22) | | |  |
| --- | --- | --- | --- | --- | --- | --- | --- | --- | --- |
|  | | *d* | lower | upper |  | *d* | lower | upper |  |
| ACI |  | 0.545 | -0.322 | 1.413 |  | 0.146 | -0.958 | 1.249 |  |
| ACI-A |  | 0.756 | 0.521 | 0.991 |  | 0.006 | -0.297 | 0.309 |  |
| ACI-B |  | 0.531 | 0.287 | 0.776 |  | 0.306 | -0.056 | 0.667 |  |
| ACI-C |  | 0.220 | -0.037 | 0.477 |  | 0.164 | -0.207 | 0.535 |  |
| ACI-D |  | 0.508 | 0.280 | 0.737 |  | 0.073 | -0.273 | 0.419 |  |

*Notes*. ACI = ADHD Credibility Index; ACI-A = Supposed Symptoms Subscale; ACI-B = Exaggerated Symptoms Subscale; ACI-C = Symptom Combinations Subscale; ACI-D = Selectivity Subscale
